# Deletion of Protocadherin Gamma C3 Induces Phenotypic and Functional Changes in Brain Microvascular Endothelial Cells *In Vitro*

**DOI:** 10.3389/fphar.2020.590144

**Published:** 2020-11-30

**Authors:** Lydia Gabbert, Christina Dilling, Patrick Meybohm, Malgorzata Burek

**Affiliations:** Department of Anaesthesia and Critical Care, University of Würzburg, Würzburg, Germany

**Keywords:** blood-brain barrier, protocadherin gamma C3, inflammation, oxygen/glucose deprivation, stroke, tumor necrosis factor-α, proliferation

## Abstract

Inflammation of the central nervous system (CNS) is associated with diseases such as multiple sclerosis, stroke and neurodegenerative diseases. Compromised integrity of the blood-brain barrier (BBB) and increased migration of immune cells into the CNS are the main characteristics of brain inflammation. Clustered protocadherins (Pcdhs) belong to a large family of cadherin-related molecules. Pcdhs are highly expressed in the CNS in neurons, astrocytes, pericytes and epithelial cells of the choroid plexus and, as we have recently demonstrated, in brain microvascular endothelial cells (BMECs). Knockout of a member of the Pcdh subfamily, PcdhgC3, resulted in significant changes in the barrier integrity of BMECs. Here we characterized the endothelial PcdhgC3 knockout (KO) cells using paracellular permeability measurements, proliferation assay, wound healing assay, inhibition of signaling pathways, oxygen/glucose deprivation (OGD) and a pro-inflammatory cytokine tumor necrosis factor alpha (TNFα) treatment. PcdhgC3 KO showed an increased paracellular permeability, a faster proliferation rate, an altered expression of efflux pumps, transporters, cellular receptors, signaling and inflammatory molecules. Serum starvation led to significantly higher phosphorylation of extracellular signal-regulated kinases (Erk) in KO cells, while no changes in phosphorylated Akt kinase levels were found. PcdhgC3 KO cells migrated faster in the wound healing assay and this migration was significantly inhibited by respective inhibitors of the MAPK-, β-catenin/Wnt-, mTOR- signaling pathways (SL327, XAV939, or Torin 2). PcdhgC3 KO cells responded stronger to OGD and TNFα by significantly higher induction of interleukin 6 mRNA than wild type cells. These results suggest that PcdhgC3 is involved in the regulation of major signaling pathways and the inflammatory response of BMECs.

## Introduction

Inflammation is one of the characteristics of CNS disorders. During ischemic stroke, increased levels of pro-inflammatory cytokines are associated with damage to the blood-brain barrier (BBB) (Hotter et al., 2019; Ittner et al., 2020). The BBB is formed by endothelial cells surrounded by pericytes, a basement membrane and astrocytes. Endothelial cells are connected by tight junctions that maintain BBB integrity and regulate paracellular transport from blood to the brain (Abbott, 2013; Wong et al., 2013).

Protocadherins (Pcdhs) with more than 80 members constitute the largest subgroup of the cadherin superfamily, which mediate calcium-dependent cell-cell adhesion. Sano et al. first discovered this large group of cadherin-related molecules with cadherin-like extracellular (EC) but distinct cytoplasmatic domains (Sano et al., 1993; Nollet et al., 2000). While classical cadherins mediate strong cell-cell adhesion, Pcdhs are both adhesive and repulsive (Phillips et al., 2017). Pcdhs can be divided into clustered Pcdhs (α-Pcdhs, β-Pcdhs, γ-Pcdhs) organized in three tandem arrays on mouse chromosome 18 and human chromosome 5, and non-clustered δ-Pcdhs, which are scattered throughout the genome (Weiner and Jontes, 2013). Pcdhs are strongly expressed in the CNS and promote neuronal development and survival. They have been described in astrocytes, pericytes, choroid plexus epithelial cells and in brain microvascular endothelial cells (BMECs) (Garrett et al., 2012; Lobas et al., 2012; Dilling et al., 2017). Pcdhs play a critical role in cell survival (Peek et al., 2017), neuronal differentiation and migration (Weiner and Jontes, 2013), synapse development (Garrett et al., 2012) and dendritic morphogenesis (Suo et al., 2012; Molumby et al., 2017). In addition, they have also been characterized in kidney, lung and colon cells and show effects on both tumor suppressive and progressive functions during malignant cell growth (Okazaki et al., 2002; Dallosso et al., 2009; Zhou et al., 2017).

γ-Pcdh gene clusters contain variable exons which encode the cadherin-like extracellular domains, the transmembrane domain and the variable cytoplasmatic domain. γ-Pcdhs also have three C-like variable exons that are expressed in neurons (Kaneko et al., 2006; Phillips et al., 2017). Mice lacking one of the γ-Pcdh-C3, C4 or C5 isoforms died due to neuronal apoptosis, similar to mice lacking all γ-Pcdhs, suggesting that these C-type-isoforms play a special role in the neuronal survival (Chen et al., 2012; Peek et al., 2017; Miralles et al., 2020).

A member of the Pcdh subfamily, PcdhgC3, is the only isoform that inhibits β-catenin/Wnt- and mTOR-signalling pathways in colorectal cancer, and is a potential tumor suppressor (Dallosso et al., 2012; Mah et al., 2016; Mah and Weiner, 2017). We recently described the expression of γ-Pcdhs in BMECs and showed that the deletion of PcdhgC3 resulted in reduced barrier integrity and changes in gene expression in BMECs (Dilling et al., 2017). Here, we hypothesized that PcdhgC3, similar to cancer cells and neurons, plays a role in multiple signaling pathways in BMECs that lead to functional changes. To investigate this, we used a BMEC PcdhgC3 KO in functional tests such as paracellular permeability measurement, proliferation, wound healing assay, response to signaling pathway inhibitors, oxygen/glucose deprivation (OGD) and TNFα. Our results help to further uncover the role of PcdhgC3 in endothelial cell biology by pointing out its importance for signaling processes at the BBB.

## Material and Methods

### Chemicals

Stock solutions of the inhibitors SL327 (Mek1/2 inhibitor, 200 nM; Sigma-Aldrich), Torin 2 (mTOR-inhibitor, 25 nM; Sigma-Aldrich) and XAV939 (selective β-catenin/Wnt pathway inhibitor, 20 μM; Sigma-Aldrich), were prepared in DMSO and diluted to the final concentration in cell culture medium. TNFα (Sigma-Aldrich) was dissolved in cell culture medium to a working concentration of 10 nM.

### Cell Culture

Mouse brain microvascular endothelial cell line, cerebEND was isolated and immortalized as previously described (Silwedel and Forster, 2006; Burek et al., 2012; Helms et al., 2016). To generate the PcdhgC3 knockout, cerebEND cells were co-transfected with Pcdh2 CRISPR/Cas9 and Pcdh2 HDR vectors (sc-430015 and sc-430015-HDR, Santa Cruz Biotechnology) using Effectene Transfection Reagent (Qiagen). The positive clones were selected with 3 µg/ml puromycin for 4 weeks (Dilling et al., 2017). The cells were grown on gelatin-coated plates in DMEM supplemented with 10% fetal calf serum (FCS). The cells were treated with 10 nM TNFα for 24 h and harvested for qPCR analysis.

### Permeability Measurement

Wild type (WT) and PcdhgC3 knockout (KO) cerebEND cells were grown on gelatin-coated transwells (pore size 0.4 µm, Corning) for 6 days. The permeability assay was performed as previously described (Curtaz et al., 2020) using 1 µM fluorescein (376 kDa, Sigma-Aldrich) for 1 h with aliquots taken from the basolateral compartment every 20 min. A parallel assay with cell-free transwells was used to calculate the endothelial permeability coefficient (Pe). The Pe of KO cells was normalized to WT cells.

### Proliferation Assay

The proliferation assay was performed using a 5′-bromo-2′-deoxyuridine (BrdU) Cell Proliferation Assay ELISA Kit (Merck) according to the manufacturer’s instructions. WT and KO cerebEND cells (5 x 10^3^ cells) suspended in 100 µL culture medium were seeded in gelatin-coated 96-well plate. After the cells attached, the BrdU Label solution was added and the cells were allowed to grow for 24 h. The absorbance was measured using a spectrophotometric plate reader (Tecan) at dual wavelengths of 450 and 540 nm.

### *In Vitro* Stroke Model, Oxygen/Glucose Deprivation

Confluent WT or KO cells were serum-starved for 24 h (1% charcoal stripped FCS). For OGD, the medium was exchanged for glucose-free DMEM and the incubation in a 1% O_2_ incubator was carried out for 4 h, as described previously (Burek et al., 2019). Normoxic control cells were only subjected to a complete medium exchange.

### Quantitative Polymerase Chain Reaction

Quantitative PCR was performed as previously described (Kaiser et al., 2018). Briefly, total RNA was isolated from cells using the Nucleospin RNA Isolation Kit (Macherey-Nagel) according to the manufacturer’s instructions. We used a High Capacity cDNA Revers Transcription Kit (Thermo Fisher Scientific) for cDNA synthesis and TaqMan Fast Advanced Master Mix in StepOnePlus Real-Time PCR System (Thermo Fisher Scientific) for quantitative PCR. Commercially available TaqMan Gene Expression Assays were used (Thermo Fisher Scientific). Calnexin and 18S-RNA were used as endogenous controls. Relative expression was calculated using the comparative Ct method.

### Western Blot

Western blot was performed as previously described (Burek et al., 2019; Curtaz et al., 2020). Primary antibodies were diluted in phosphate-buffered saline (PBS) containing 1% bovine serum albumin (BSA). The following primary antibodies were used: rat anti-Bcrp (1:1,000, Abcam #Ab-24114), mouse anti-Glut-1 (1:200, Millipore #07–1401), rabbit anti-Lrp1 (1:1,000, Abcam #Ab92544), mouse anti-Mrp1 (1:1,000, Millipore #MAB4100), rat anti-Mrp4 (1:1,000, Enzo Life Science #ALX-801–039-C100), mouse anti-Tfrc (Transferrin Receptor, 1:500, Thermo Fisher Scientific #13–6,800), goat anti-RAGE (1:200, Santa Cruz #sc-8230), rabbit anti-PcdhgC3 (1: 10,000 (Frank et al., 2005)), mouse anti-p44/42 MAPK (Erk1/2) (1:2,000, Cell Signaling #9107), rabbit anti-phospho-Erk1/2 (1:2,000, Cell Signaling #4370), rabbit anti-Akt (1:1,000, Cell Signaling #9272) and rabbit anti-phospho Akt (1:1,000, Cell Signaling #4058). After incubation with respective secondary antibodies, images were taken using an Enhanced Chemiluminescence solution and FluorChem FC2 Multi-Imager II (Alpha Innotech). The intensity of the protein bands was estimated using the ImageJ software.

### Enzyme-Linked Immunosorbent Assay

The cell culture medium was collected and kept frozen at −80°C until use. Ccl2/Mcp-1 and Ccl5/RANTES Quantikine ELISA Kit (R&D Systems) were performed according to the manufacturer’s protocol.

### Wound Healing Assay

The wound healing assay was performed as previously described (Blecharz-Lang et al., 2018a). Briefly, cells were seeded on µ-Dish (Ibidi GmbH) and grown to confluence. After 24 h of serum starvation, the cells were left untreated or were treated for 48 h with MAPK-, β-catenin/Wnt-, mTOR-signaling pathway inhibitors (SL327, XAV939, and Torin 2) in triplicates. The wells separating the cells were removed and the dishes were photographed at time 0 h. The cells were allowed to grow in a 500-µm-space for 48 h and were photographed again at 48 h using a Keyence BZ9000 microscope (Keyence). The difference in area covered between 0 and 48 h was calculated and a migration rate was normalized to the control, which was set arbitrarily to 1.

### Statistical Analysis

GraphPad Prism 7 (GraphPad Software) was used for statistical analysis. The data are expressed as the mean ± standard deviation. Unpaired *t* test was used to compare two groups while two-way ANOVA with Tukey’s multiple comparison test was used to compare WT and KO cells with or without treatment. Statistical significance was assumed at *p* < 0.05 (*).

## Results

### C-Terminal Truncated Fragment of PcdhgC3 Shows Higher Level in Confluent Brain Microvascular Endothelial Cells

**FIGURE 1:**
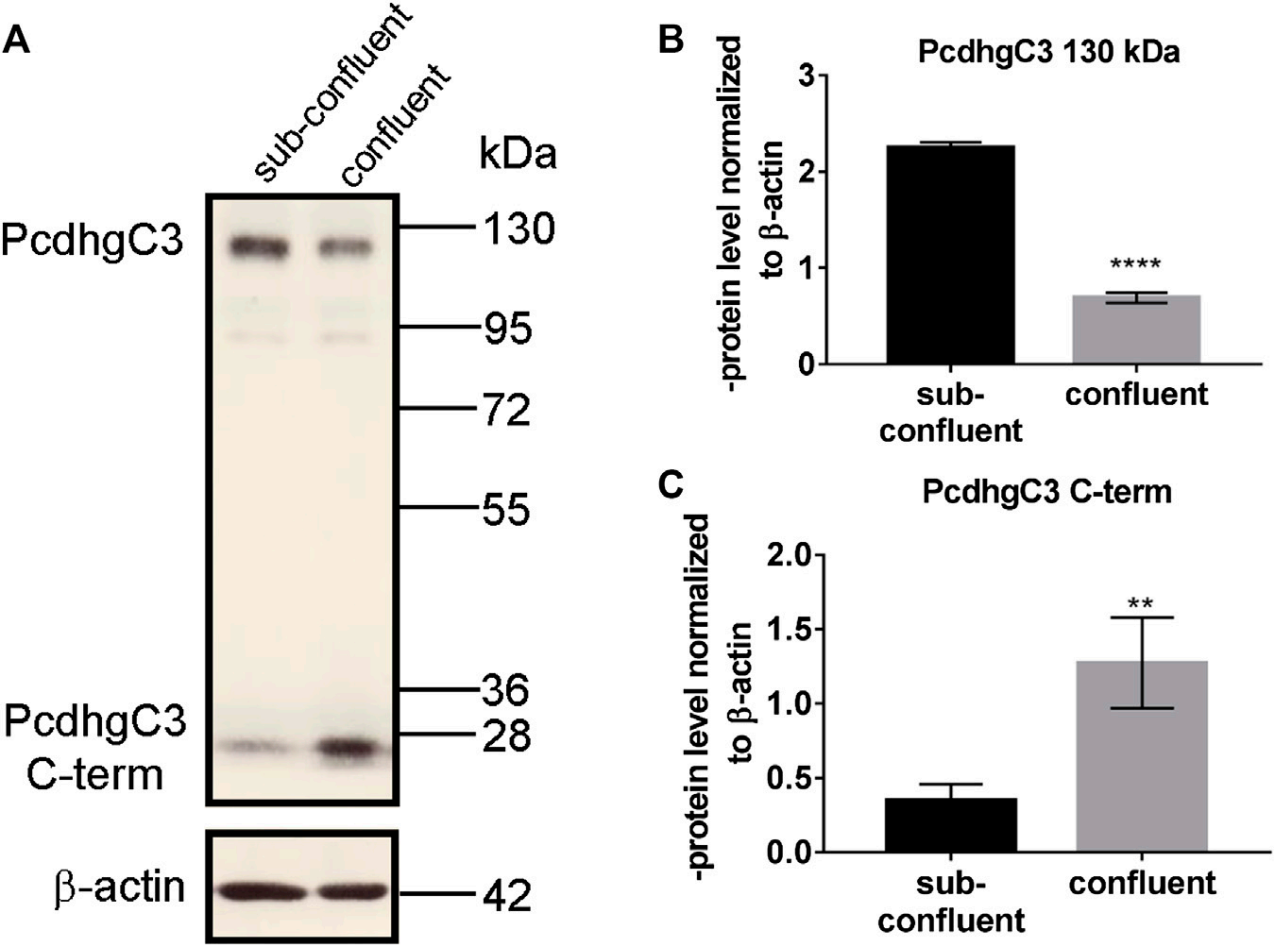
C-terminal fragment of PcdhgC3 accumulates in confluent Brain Microvascular Endothelial Cells. BMECs were seeded on gelatin-coated plates and were grown for 2 or 7 days followed by protein extraction and Western blot. PcdhgC3 protein level was analyzed by Western blot **(A)** and the bands corresponding to the full-length protein (130 kDa) **(B)** and truncated C-terminal fragment of PcdhgC3 (25 kDa) **(C)** were analyzed by densitometry. Data are shown as mean ± standard deviation of three independent experiments. **p < 0.01, ****p < 0.0001.

The C-terminus of γ-Pcdhs is cleaved by presenilin and the resulting intracellular fragment can be localized to the nucleus and have potential signaling functions (Haas et al., 2005). Interestingly, the 25 kDa C-terminal truncated fragment of PcdhgC3 is enriched in confluent BMECs and shows significantly lower level in non-confluent BMECs, which is opposite to the full-length protein (Figures 1A–C). Confluent BMECs build a tight monolayer. This is accompanied by multiple changes in gene and protein expression and intracellular signal transmission. Pcdhs play a role in contact inhibition of cell proliferation and are potential candidates for tumor suppressors (Okazaki et al., 2002; Yu et al., 2008). The increase in the cleaved PcdhgC3 fragment specifically in confluent BMECs suggests a distinct role for this fragment in processes such as cell proliferation, but the exact mechanisms in BMECs have yet to be investigated.

### PcdhgC3 Knockout Cells Show Increased Paracellular Permeability, Proliferation and Changes in Protein and Gene Expression

**FIGURE 2:**
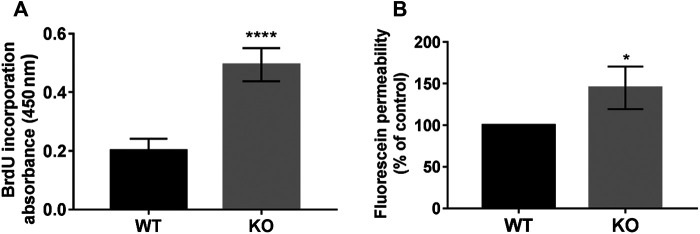
PcdhgC3 knockout cells show increased proliferation and paracellular permeability. **(A)** The proliferation rate of WT and KO cells was measured using BrdU ELISA Kit at 16 h. **(B)** Wild type (WT) and PcdhgC3 knockout (KO) cells were grown in transwells for 6 days followed by measuring the paracellular permeability for fluorescein. Paracellular permeability of KO cells was normalized to WT cells, which was set as 100%. Data are shown as mean ± standard deviation of three independent experiments. *p < 0.05, ****p < 0.0001.

**FIGURE 3:**
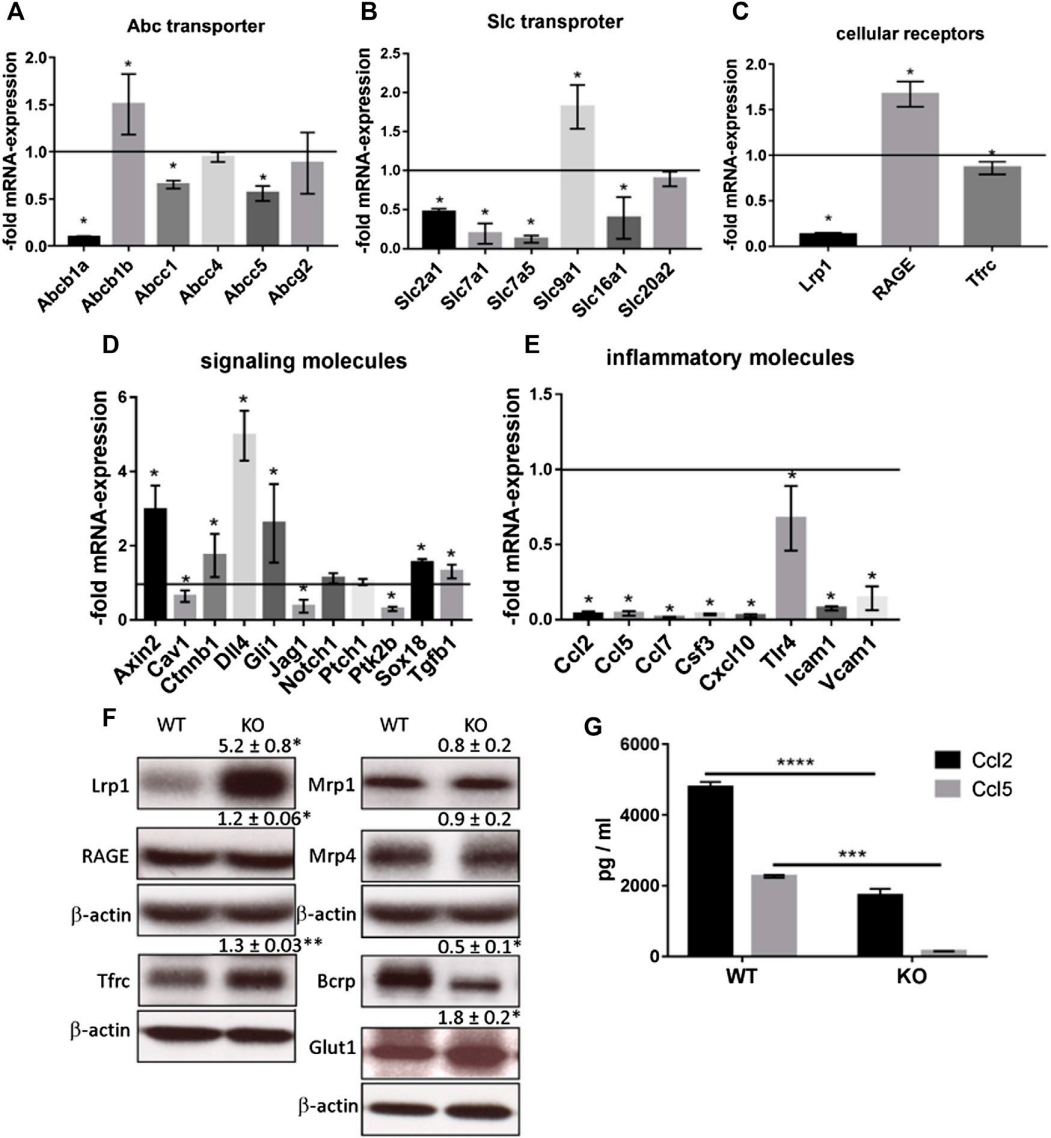
Brain Microvascular Endothelial Cells lacking PcdhgC3 show differential gene and protein expression. Wild type and PcdhgC3 knockout BMECs were grown to confluence, serum-starved for 24 h and harvested for RNA or protein extraction. Target gene expression of Abc transporter **(A)**, Slc transporter **(B)**, cellular receptors **(C)**, signaling molecules **(D)** and inflammatory molecules **(E)**, was normalized to endogenous control and shown as fold over control (wild type cells), which was arbitrarily set as 1 (control level marked in graph). **(F)** Protein expression of selected Abc transporter (Bcrp, Mrp1, Mrp4), Slc transporter (Glut1), cellular receptors (Lrp1, RAGE, Tfrc) was analyzed by Western blot. Protein expression was normalized to β-actin and to WT cells. The densitometry values are given above the corresponding bands. **(G)** Protein levels of Ccl2 and Ccl5 were analyzed by ELISA in cell culture medium of WT and KO cells. Data are shown as mean ± standard deviation of three independent experiments. *p < 0.05, **p < 0.01, ***p < 0.001, ****p < 0.0001. Abbreviations **(A)** Abcb1a: P-Glycoprotein, ATP binding cassette subfamily B member 1A, Abcb1b: P-Glycoprotein, ATP binding cassette subfamily B member 1, Abcc1: Mrp1, Multidrug resistance associated protein 1, Abcc4: Mrp4, Multidrug resistance associated protein 4, Abcc5: ATP binding cassette subfamily C member 5, Abcg2: BCRP, Breast cancer resistance protein **(B)** Slc2a1: Solute carrier family 2 member 1, Glut1, Glucose transporter type 1, Slc7a1: Solute carrier family 7 member 1, Cat1, Cationic amino acid transporter 1, Slc7a5: Solute carrier family 7 member 5, Lat1, L-Type amino acid transporter 1), Slc9a1: Solute carrier family 9 member 1, Nhe1, Cation proton antiporter 1, Slc16a1: Solute carrier family 16 member 1, Mct1, Monocarboxylate transporter 1, Slc20a2: Solute carrier family 20 member 2, Pit2, Phosphate transporter 2 **(C)** Lrp1: Low density lipoprotein receptor-related protein 1, RAGE: Receptor for Advanced Glycosylation End Products, Tfrc: Transferrin receptor **(D)** Cav1: Caveolin 1, Ctnnb1: Catenin beta 1, Dll4: Delta like canonical Notch ligand 4, Gli1: Family zinc finger 1, Jag1: Jagged canonical Notch ligand 1, Notch1: Notch receptor 1, Ptch1: Patched 1, Ptk2b: Protein tyrosine kinase 2 beta, Sox18: SRY-Box transcription factor 18, Tgfb1: Transforming growth factor beta 1 **(E)** Ccl2, 5, 7: C-C motif chemokine ligand 2, 5,7, Csf3: Colony stimulating factor 3, Cxcl10: C-X-C motif chemokine ligand 10, Tlr4: Toll like receptor 4, Icam1: Intercellular adhesion molecule 1, Vcam1: Vascular cell adhesion molecule 1.

**FIGURE 4:**
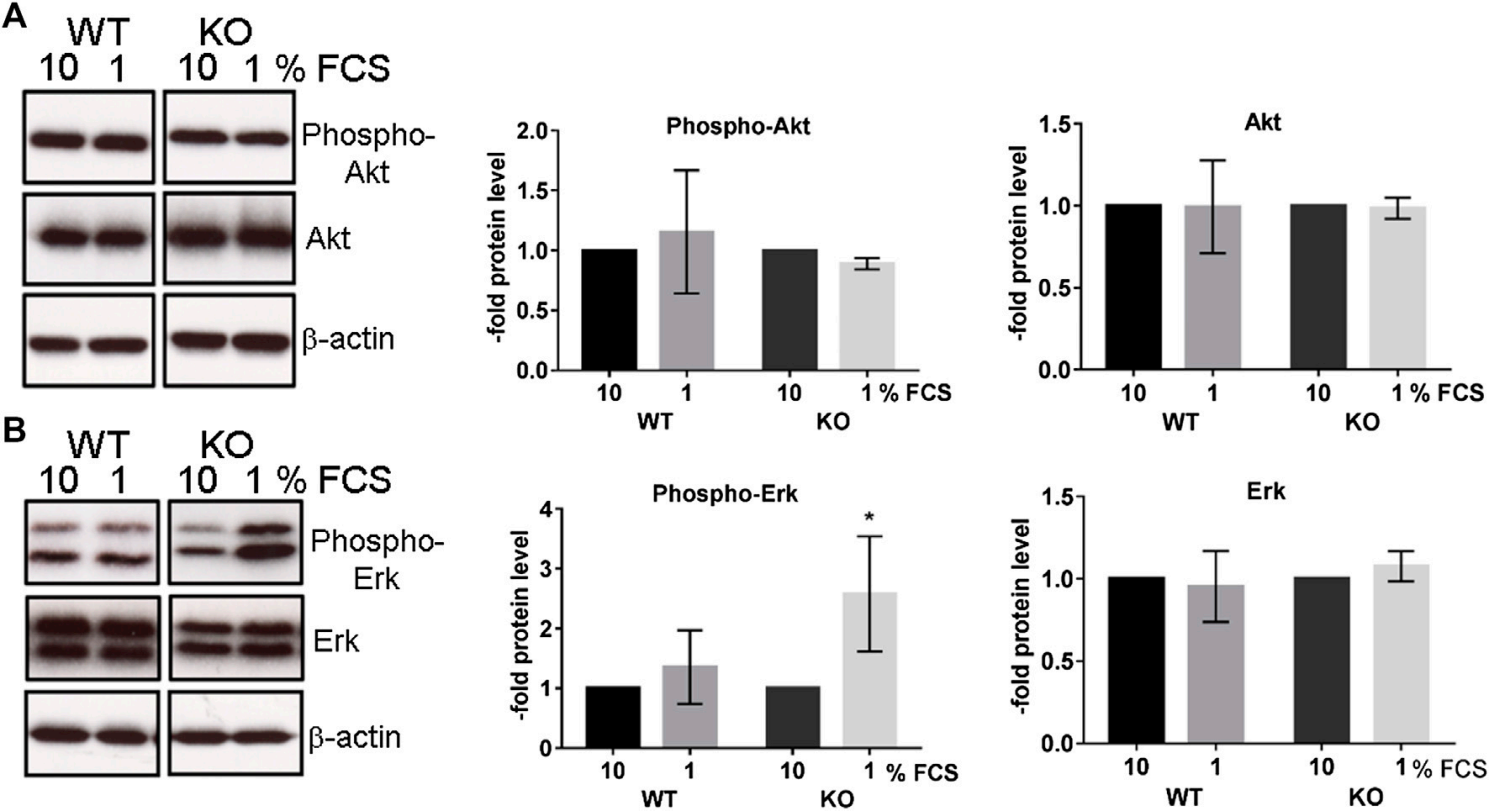
Serum-starved PcdhgC3 knockout cells show higher phosphorylation of extracellular signal-regulated kinases (Erk). Wild type (WT) and PcdhgC3 knockout (KO) BMECs were grown to confluence, serum-starved (1% FCS) or not (10% FCS) for 24 h and harvested for protein extraction and Western blot analysis. **(A)** Phosphorylated (Phospho-Akt) and total Akt **(B)** phosphorylated Erk (Phospho-Erk) and total Erk were detected with specific antibodies. The protein levels were analyzed by densitometry. Data are shown as mean ± standard deviation of three independent experiments. *p < 0.05.

We measured and compared the proliferation rate of WT and KO cells by BrdU incorporation into newly synthesized DNA (Figure 2A **)**. The PcdhgC3 KO cells proliferated 2.46 ± 0.1 fold faster than the WT cells, suggesting the role of PcdhgC3 in endothelial cell proliferation. Increased proliferation leads to more dedifferentiated phenotype and lower barrier properties. We therefore measured the paracellular permeability for fluorescein in WT and KO cells (Figure 2B). Consistent with previous results (Dilling et al., 2017), PcdhgC3 KO cells showed an increase in permeability of 45% ± 14.7 compared to WT cells (Figure 2B). Next, we tested the effects of PcdhgC3 KO on gene expression of endothelial and BBB markers as well as on genes involved in inflammatory and signaling pathways (Figure 3). A comparison of gene expression between WT and KO cells showed increased expression of the efflux pump Abcb1b (P-Glycoprotein, ATP Binding Cassette Subfamily B Member 1) and a significantly reduced Abcb1a (P-Glycoprotein, ATP binding cassette subfamily B member 1a), Abcc1 (Mrp1, Multidrug resistance associated protein 1) and Abcc5 (ATP binding cassette subfamily C member 5) expression (Figure 3A). Among the solute carrier transporters analyzed, the Slc9a1 (Nhe1, Cation proton antiporter 1) was significantly increased, while Slc2a1 (Glucose transporter type 1), Slc7a1 (Cat1, Cationic amino acid transporter 1), Slc7a5 (Lat1, L-Type amino acid transporter 1) and Slc16a1 (Mct1, Monocarboxylic acid transporter 1) were downregulated (Figure 3B). Among the cellular receptors, PcdhgC3 KO showed increased RAGE (Receptor for Advanced Glycosylation End Products) level and decreased Lrp1 (Low-density lipoprotein receptor-related protein 1) and Tfrc (Transferrin receptor) expression (Figure 3C). Transcription factors such as Sox18 (SRY-Box transcription factor 18) and Tgfb1 (Transforming growth factor beta 1) were significantly increased in PcdhgC3 KO (Figure 3D). Genes involved in the β-catenin/Wnt-signaling pathway, Axin2 and Ctnnb1 (Catenin Beta 1) were upregulated, similar to genes involved in the Notch-signaling pathway (Dll4, Delta like canonical Notch ligand 4) and the sonic hedgehog signal transduction cascade (Gli1, Gli Family zinc finger 1) (Figure 3D). Interestingly, all of the inflammatory mediators tested, Ccl2 (C-C motif chemokine ligand 2), Ccl5 (C-C motif chemokine ligand 5), Ccl7 (C-C motif chemokine ligand 7), Csf3 (Colony stimulating factor 3), Cxcl10 (C-X-C motif chemokine ligand 10), Tlr4 (Toll like receptor 4) were downregulated in PcdhgC3 KO cells (Figure 3E). We analyzed the protein levels of selected transporter and cellular receptors (Figure 3F). We detected a significant increase in Lrp1, RAGE, Tfrc and Glut1 proteins. This was opposite to the corresponding mRNA level in KO cells. The Bcrp protein level was significantly downregulated in PcdhgC3 KO cells. Ccl2 and Ccl5 showed a decreased protein level consistently with the mRNA level (Figure 3G). Next, we tested the activation of the signaling pathways using antibodies against phosphorylated active kinases Akt and Erk (Figure 4). We used the serum starvation of the cells as a trigger for the activation of the signaling pathways. The WT and KO cells were harvested 24 h after serum reduction. Under these conditions, Akt showed no differences between WT and KO cells (Figure 4A), while significantly increased phospho-Erk level was detected in PcdhgC3 KO cells, which indicates a strong activation of this signaling pathway (Figure 4B).

### Knockout of PcdhgC3 Leads to a Higher Migration Rate of Brain Microvascular Endothelial Cells, Which Is Mediated by MAPK-, β-Catenin/Wnt- and mTOR-Signaling Pathways

**FIGURE 5:**
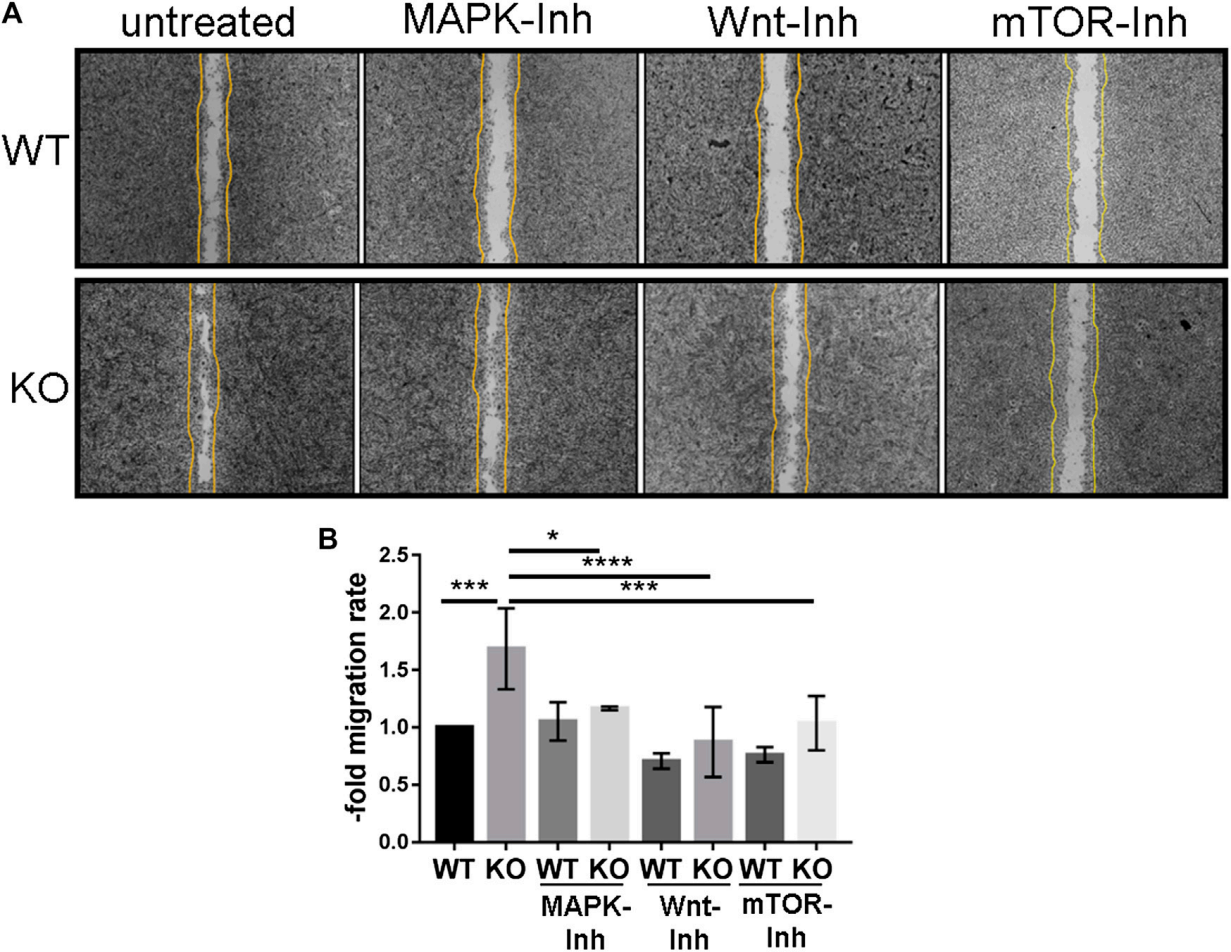
PcdhgC3 knockout Brain Microvascular Endothelial Cells migrate faster in wound healing assay and their migration can be inhibited by specific MAPK-, β-catenin/Wnt- and mTOR-signaling pathway inhibitors. Wild type (WT) and PcdhgC3 knockout (KO) BMECs were seeded on µ-Dish and were grown to confluence. After 24 h serum starvation, the cells were left untreated or were treated with MAPK- (SL327, Mek1/2 inhibitor, 200 nM), Wnt- (XAV939, selective β-catenin/Wnt pathway inhibitor, 20 µM), mTOR- (Torin 2, mTOR-inhibitor, 25 nM) signaling pathways inhibitors (MAPK-Inh, Wnt-Inh, mTOR-Inh). The wells separating the cells were removed and the dishes were photographed at time 0 h. The cells were allowed to grow for 48 h into a 500-µm-space and were photographed again at 48 h. **(A)** The difference in area covered between 0 and 48 h was calculated and a migration rate was normalized to the control, which was arbitrarily set as 1. **(B)** Data are shown as mean ± standard deviation of three independent experiments. *p < 0.05, **p < 0.01.

The increased activation of Erk in PcdhgC3 KO cells indicates the activation of signaling pathways that are involved in cell proliferation, differentiation, aging and survival (Steelman et al., 2011). We therefore tested the migration rate of WT and KO cells in a wound healing assay (Figure 5). KO cells migrated significantly faster than the WT cells (Figures 5A,B). This faster migration of KO cells was blocked by MAPK-, β-catenin/Wnt- and mTOR-pathways inhibitors. KO cells treated with inhibitors of the key signaling pathways showed a significantly lower migration rate compared to untreated KO cells (Figure 5B).

### PcdhgC3 KO Cells Respond Stronger to Oxygen/Glucose Deprivation and to Tumor Necrosis Factor Alpha Treatment

**FIGURE 6:**
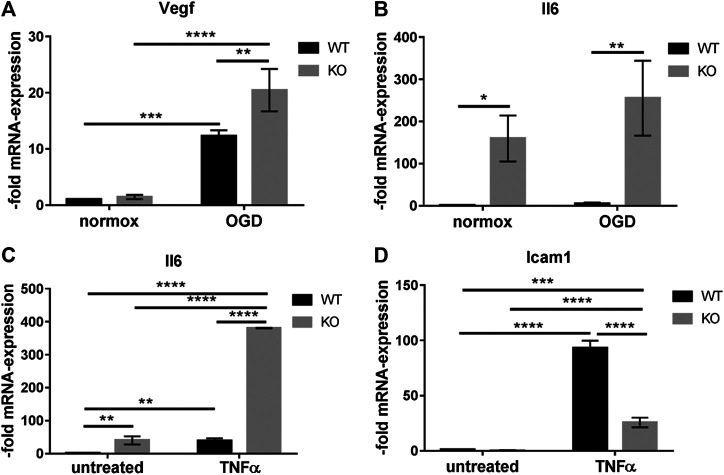
PcdhgC3 knockout Brain Microvascular Endothelial Cells respond stronger to oxygen/glucose deprivation (OGD) conditions and to Tumor Necrosis Factor Alpha treatment. Confluent wild type (WT) and PcdhgC3 knockout (KO) BMECs were subjected to OGD **(A,B)** or TNFα treatment **(C,D)** followed by RNA extraction and qPCR analysis of indicated markers. Target gene expression was normalized to endogenous control and shown as fold over control (untreated WT cells for TNFα and normoxic WT cells for OGD), which was arbitrarily set as 1. Data are shown as mean ± standard deviation of three independent experiments. Icam1, Intracellular adhesion molecule; Il6, Interleukin 6; TNFα, Tumor necrosis factor alpha; Vegf, Vascular endothelia growth factor. *p < 0.05, **p < 0.01, ***p < 0.001, ****p < 0.0001.

Increased activation of the MAPK-signaling pathway is observed in stroke, Alzheimer’s disease and multiple sclerosis. It contributes to decreased BBB integrity and production of pro-inflammatory mediators in the brain (Abbott et al., 2010; Maddahi and Edvinsson, 2010). We tested the response of WT and KO BMECs to OGD treatment. The cells were kept under OGD for 4 h (Figures 6A,B). The induction of Vegf in WT and KO cells after 4 h of OGD confirmed our experimental OGD settings, since Vegf is known as a hypoxic marker at the BBB (Zhang et al., 2000). The induction of Vegf was significantly higher in KO cells than in WT cells (Figure 6A). OGD treatment resulted in an increased Il6 expression in WT and KO cell and the increase was significantly higher in KO than in WT cells (Figure 6B). Next, we tested the response to TNFα treatment of WT and KO cells (Figures 6C,D). TNFα treatment led to the induction of Il6 (Figure 6C) and Icam1 (Figure 6D). The induction of Il6 was higher in KO cells, while the induction of Icam1 was lower in KO cells compared to WT cells. Thus, the deletion of PcdhgC3 alters the inflammatory response of BMECs suggesting a central role of PcdhgC3 in the vascular inflammatory phenotype.

## Discussion

The high expression of Pcdhs in BMECs suggests their specific intracellular role in vascular endothelial cells. Here, we further characterized the role of a member of the Pcdh subfamily, PcdhgC3, by showing its involvement in intracellular signaling and the inflammatory response.

The C-terminus of Pcdhs could have a cell surface receptor signaling function regulated by proteolytic events (Haas et al., 2005). We show here that the truncated C-terminus of PcdhgC3 is enriched in confluent BMECs. This is in line with reports showing a tumor suppressor role of Pcdhs, as they play a role in the contact inhibition of cell proliferation (Okazaki et al., 2002; Yu et al., 2008). This fragment could be responsible for signal transduction in confluent BMECs, but the exact regulation mechanism must be determined in further experiments using e.g., specific inhibitors of presenilin or PcdhgC3 mutants and their effect on signaling cascades in BMECs. One of the possible mechanisms could be the interference of PcdhgC3 with VE-cadherin/β-catenin signaling pathway, which has been shown to inhibit endothelial proliferation (Lampugnani et al., 2003). This assumption is strengthened by the observation that PcdhgC3 KO cells have an increased proliferation and a high paracellular permeability compared to WT cells. In addition, we have previously shown that PcdhgC3 KO cells have a lower TEER and a strongly decreased expression of occludin (Dilling et al., 2017). Increased proliferation and permeability of the endothelium are hallmarks of many diseases of the CNS (Daneman, 2012).

The PcdhgC3 KO BMECs showed differences in the expression level of cellular receptors. Changing the expression of cellular receptors could be a useful target for drug delivery to the brain. Drug delivery strategies that use anti-transferrin receptor antibodies have shown increased drug brain penetration in models of neurodegenerative diseases and brain tumors (Lajoie and Shusta, 2015; Wang et al., 2015). Trfc is decreased at mRNA and increased at protein level in KO cells. We observed a significantly increased RAGE mRNA and slightly but significantly increased protein level in KO cells. This receptor for advanced glycation end products plays a role in the transport of molecules such as amyloid-beta peptides through the BBB. The inhibition of RAGE-ligand interaction protects against the accumulation of amyloid-beta peptides in the brain (Deane et al., 2003). PcdhgC3 KO led to changes in the expression of the efflux pumps genes. Most of them were decreased in KO BMECs at mRNA and protein level as shown for Bcrp. Efflux pumps protect the brain from blood-borne substances by pumping them out of endothelial cells. However, many cancer drugs are also substrates of efflux pumps (Lajoie and Shusta, 2015). Glut1 protein and mRNA were inversely regulated in PcdhgC3 KO cells (mRNA was decreased while protein was increased). Increased Glut1 protein level corresponds to increased Vegf level in KO cells (Sanchez-Elsner et al., 2001). We also examined the mRNA expression of various signaling molecules. Dll4, which is involved in Notch signaling, was strongly increased. Based on other studies, the downregulation of Dll4 was accompanied by stimulated endothelial proliferation, migration and sprouting (You et al., 2013). However, our results show otherwise, but we only measured the mRNA expression of Dll4. On the other hand, decreased Dll4 expression correlated with the inhibition of the Src/Akt/β-catenin-signaling pathway in HUVEC (Human Umbilical Vein Endothelial Cells) and the Src/Akt/β-catenin-signaling pathway also directly regulates the Dll4 (Fournier et al., 2020). In addition, the mRNA of Protein tyrosine kinase PYK2 (Ptk2b) is strongly downregulated in PcdhgC3 KO, but it has been shown that this protein positively regulates the MAPK pathway and endothelial cell migration (Lev et al., 1995; Evans et al., 2011). We analyzed several genes involved in the inflammatory response. Most of the genes were downregulated. Ccl2 and -5 were additionally downregulated at the protein level, as shown by ELISA. Ccl2, -5, -7 and Icam1, which are downregulated in PcdhgC3 KO cells show positive correlation with Erk cascade (Dragoni et al., 2017). Since Erk-pathway is upregulated in our case, an opposite regulation of Ccl-2, -5, -7 and Icam1 could be expected. However, we show that due to the deletion of PcdhgC3, other signaling pathways are also deregulated. The effects could be additive and therefore not correlate with the literature. In addition, the activation of Erk in KO cells is not constitutive, as shown by phospho-antibodies, but requires a trigger such as the serum starvation shown here.

In contrast to WT cells, KO cells showed activation of Erk-signaling pathway in response to serum starvation and growth factor reduction. Under normal culture conditions (10% FCS), WT and KO cells do not differ in phosho-Erk level. This indicates that PcdhgC3 may have a protective function in BMECs under stress condition. The increased activity of MAPK-signaling pathway can affect the migration rate of BMECs in wound healing assay. The PcdhgC3 KO cells migrated significantly faster than the WT cells. This increased migration rate was mediated in part by the activity of MAPK-, β-catenin/Wnt- or mTOR-signaling pathways, as demonstrated by the addition of selective inhibitors. Our study is however, limited, since we only analyzed the Erk-activation after 24 h. This activation could be time dependent. Additionally, we can only assume that downstream signaling molecules of the Erk signaling pathway are also activated. The regulation of signaling pathways and the inhibition of cell proliferation has previously been shown in different types of cancer for different Pcdhs (Dallosso et al., 2012; Zhou et al., 2017). The mTOR pathway also plays a role in endothelial cell proliferation. In coronary heart disease as well as in hemangiomas, inhibition of the mTOR pathway blocked abnormal endothelial cell proliferation (Wang et al., 2017; Harari et al., 2018). Endothelial cell proliferation and migration also occurs in the brain during inflammation and hypoxia. Meanwhile, the MAPK-signaling pathway activates pro-inflammatory mediators that contribute to BBB damage in stroke, traumatic brain injury and dementia (Maddahi and Edvinsson, 2010; Zhu et al., 2018; Griemert et al., 2019). PcdhgC3 KO is more responsive to OGD and to TNFα treatment. PcdhgC3 KO showed a higher basal level and induction of the pro-inflammatory cytokine Il6 and Vegf than the WT cells after OGD and/or TNFα treatment. Also, the Icam1 expression was lower in KO cells and its induction by TNFα was lower than in WT cells. This correlated with the low level of other inflammatory molecules in KO cells, which was shown as evidenced by qPCR and ELISA. BMECs express Il6 and TNFα receptors, secrete Il6 and TNFα and can transmit their intracellular signaling (Coisne and Engelhardt, 2011; Blecharz-Lang et al., 2018b). Expression of Il6 is low in the normal brain and dramatically increases in response to an inflammatory stimulus such as TNFα (Shalaby et al., 1989; Juttler et al., 2002). Elevated level of Il6 could lead to greater damage to neuronal tissue during hypoxic events in the brain. A high Il6 level in PcdhgC3 KO cells corresponds to a high Vegf level and an activated Erk-signaling pathway. Consistent with our results, Il6 has been shown to trigger endothelial cell proliferation by increasing Vegf release and activating Erk-signaling (Yao et al., 2006). Inflammation mediators directly influence junctional proteins of the BBB such as claudin-5 and ZO-1 leading to further functional changes (Burek and Forster, 2009; Ittner et al., 2020).

The phenotypic changes described here are only shown *in vitro* in BMECs without the influence of other cell types present *in vivo* in the brain. This is a major limitation of our study. Nonetheless, we plan to generate endothelial-specific knockout mice of PcdhgC3 in order to investigate the role of PcdhgC3 *in vivo* in brain vascular endothelial cells.

To summarize, our data show that the deletion of only one member of the Pcdh subfamily in BMECs can lead to multiple phenotypic and functional changes. Loss of PcdhgC3 can switch cells from resting barrier type to a more migratory/proliferating and angiogenic type. We provide the first evidence for PcdhgC3-mediated regulation of signaling pathways in BMECs and a changed inflammatory or hypoxia/hypoglycemia response.

## Data Availability Statement

The raw data supporting the conclusions of this article will be made available by the authors, without undue reservation, to any qualified researcher.

## Author Contributions

LG, CD, and MB designed the study, performed and analyzed experiments and drafted the manuscript. PM provided crucial reagents. All authors were involved in critical revision and final approval of the manuscript.

## Funding

This publication was supported by the Open Access Publication Fund of the University of Wurzburg and institutional funds of the Unviersity Hospital Wurzburg.

## Conflict of Interest

The authors declare that the research was conducted in the absence of any commercial or financial relationships that could be construed as a potential conflict of interest.

## References

[B1] Abbott N. J. (2013). Blood-brain barrier structure and function and the challenges for CNS drug delivery. J. Inherit. Metab. Dis. 36, 437–449. 10.1007/s10545-013-9608-0. 23609350

[B2] Abbott N. J. Patabendige A. A. K. Dolman D. E. M. Yusof S. R. Begley D. J. (2010). Structure and function of the blood-brain barrier. Neurobiol. Dis. 37, 13–25. 10.1016/j.nbd.2009.07.030. 19664713

[B3] Blecharz-Lang K. G. Prinz V. Burek M. Frey D. Schenkel T. Krug S. M. (2018a). Gelatinolytic activity of autocrine matrix metalloproteinase-9 leads to endothelial de-arrangement in Moyamoya disease. J. Cereb. Blood Flow Metab. 38, 1940–1953. 10.1177/0271678x18768443. 29633884 PMC6259317

[B4] Blecharz-Lang K. G. Wagner J. Fries A. Nieminen-Kelhä M. Rösner J. Schneider U. C. (2018b). Interleukin 6-mediated endothelial barrier disturbances can Be attenuated by blockade of the IL6 receptor expressed in brain microvascular endothelial cells. Transl. Stroke Res. 9, 631–642. 10.1007/s12975-018-0614-2. 29429002

[B5] Burek M. Salvador E. Förster C. Y. (2012). Generation of an immortalized murine brain microvascular endothelial cell line as an *in vitro* blood brain barrier model. J. Vis. Exp., e4022 10.3791/4022. 22951995 PMC3486758

[B6] Burek M. Förster C. Y. (2009). Cloning and characterization of the murine claudin-5 promoter. Mol. Cell. Endocrinol. 298, 19–24. 10.1016/j.mce.2008.09.041. 18996436

[B7] Burek M. König A. Lang M. Fiedler J. Oerter S. Roewer N. (2019). Hypoxia-Induced MicroRNA-212/132 alter blood-brain barrier integrity through inhibition of tight junction-associated proteins in human and mouse brain microvascular endothelial cells. Transl. Stroke Res. 10, 672–683. 10.1007/s12975-018-0683-2. 30617994 PMC6842347

[B8] Chen W. V. Alvarez F. J. Lefebvre J. L. Friedman B. Nwakeze C. Geiman E. (2012). Functional significance of isoform diversification in the protocadherin gamma gene cluster. Neuron 75, 402–409. 10.1016/j.neuron.2012.06.039. 22884324 PMC3426296

[B9] Coisne C. Engelhardt B. (2011). Tight junctions in brain barriers during central nervous system inflammation. Antioxid. Redox Signal. 15, 1285–1303. 10.1089/ars.2011.3929. 21338320

[B10] Curtaz C. J. Schmitt C. Herbert S. L. Feldheim J. Schlegel N. Gosselet F. (2020). Serum-derived factors of breast cancer patients with brain metastases alter permeability of a human blood-brain barrier model. Fluids Barriers CNS 17, 31 10.1186/s12987-020-00192-6. 32321535 PMC7178982

[B11] Dallosso A. R. Hancock A. L. Szemes M. Moorwood K. Chilukamarri L. Tsai H. H. (2009). Frequent long-range epigenetic silencing of protocadherin gene clusters on chromosome 5q31 in Wilms’ tumor. PLoS Genet. 5, e1000745 10.1371/journal.pgen.1000745. 19956686 PMC2776977

[B12] Dallosso A. R. Øster B. Greenhough A. Thorsen K. Curry T. J. Owen C. (2012). Long-range epigenetic silencing of chromosome 5q31 protocadherins is involved in early and late stages of colorectal tumorigenesis through modulation of oncogenic pathways. Oncogene 31, 4409–4419. 10.1038/onc.2011.609. 22249255 PMC3647107

[B13] Daneman R. (2012). The blood-brain barrier in health and disease. Ann. Neurol. 72, 648–672. 10.1002/ana.23648. 23280789

[B14] Deane R. Du Yan S. Submamaryan R. K. LaRue B. Jovanovic S. Hogg E. (2003). RAGE mediates amyloid-β peptide transport across the blood-brain barrier and accumulation in brain. Nat. Med. 9, 907–913. 10.1038/nm890. 12808450

[B15] Dilling C. Roewer N. Förster C. Y. Burek M. (2017). Multiple protocadherins are expressed in brain microvascular endothelial cells and might play a role in tight junction protein regulation. J. Cereb. Blood Flow Metab. 37, 3391–3400. 10.1177/0271678x16688706. 28094605 PMC5624389

[B16] Dragoni S. Hudson N. Kenny B.-A. Burgoyne T. McKenzie J. A. Gill Y. (2017). Endothelial MAPKs direct ICAM-1 signaling to divergent inflammatory functions. J. Immunol. 198, 4074–4085. 10.4049/jimmunol.1600823. 28373581 PMC5421301

[B17] Evans I. M. Yamaji M. Britton G. Pellet-Many C. Lockie C. Zachary I. C. (2011). Neuropilin-1 signaling through p130Cas tyrosine phosphorylation is essential for growth factor-dependent migration of glioma and endothelial cells. Mol. Cell Biol. 31, 1174–1185. 10.1128/mcb.00903-10. 21245381 PMC3067908

[B18] Fournier P. Viallard C. Dejda A. Sapieha P. Larrivée B. Royal I. (2020). The protein tyrosine phosphatase PTPRJ/DEP-1 contributes to the regulation of the Notch-signaling pathway and sprouting angiogenesis. Angiogenesis 23, 145–157. 10.1007/s10456-019-09683-z. 31598898

[B19] Frank M. Ebert M. Shan W. Phillips G. R. Arndt K. Colman D. R. (2005). Differential expression of individual gamma-protocadherins during mouse brain development. Mol. Cell. Neurosci. 29, 603–616. 10.1016/j.mcn.2005.05.001. 15964765

[B20] Garrett A. M. Schreiner D. Lobas M. A. Weiner J. A. (2012). γ-Protocadherins control cortical dendrite arborization by regulating the activity of a FAK/PKC/MARCKS signaling pathway. Neuron 74, 269–276. 10.1016/j.neuron.2012.01.028. 22542181 PMC3582349

[B21] Griemert E. V. Schwarzmaier S. M. Hummel R. Gölz C. Yang D. Neuhaus W. (2019). Plasminogen activator inhibitor‐1 augments damage by impairing fibrinolysis after traumatic brain injury. Ann. Neurol. 85, 667–680. 10.1002/ana.25458. 30843275 PMC6593843

[B22] Haas I. G. Frank M. Veron N. Kemler R. (2005). Presenilin-dependent processing and nuclear function of -protocadherins. J. Biol. Chem. 280, 9313–9319. 10.1074/jbc.m412909200. 15611067

[B23] Harari E. Guo L. Smith S. L. Paek K. H. Fernandez R. Sakamoto A. (2018). Direct targeting of the mTOR (mammalian target of rapamycin) kinase improves endothelial permeability in drug-eluting stents-brief report. Arterioscler. Thromb. Vasc. Biol. 38, 2217–2224. 10.1161/atvbaha.118.311321. 30026269

[B24] Helms H. C. Abbott N. J. Burek M. Cecchelli R. Couraud P.-O. Deli M. A. (2016). *In vitro* models of the blood-brain barrier: an overview of commonly used brain endothelial cell culture models and guidelines for their use. J. Cereb. Blood Flow Metab. 36, 862–890. 10.1177/0271678x16630991. 26868179 PMC4853841

[B25] Hotter B. Hoffmann S. Ulm L. Meisel C. Fiebach J. B. Meisel A. (2019). IL-6 plasma levels correlate with cerebral perfusion deficits and infarct sizes in stroke patients without associated infections. Front. Neurol. 10, 83 10.3389/fneur.2019.00083. 30828313 PMC6384225

[B26] Ittner C. Burek M. Stork S. Nagai M. Forster C. Y. (2020). Increased catecholamine levels and inflammatory mediators alter barrier properties of brain microvascular endothelial cells *in vitro* . Front. Cardiovasc. Med. 7, 73 10.3389/fcvm.2020.00073. 32432126 PMC7214675

[B27] Jüttler E. Tarabin V. Schwaninger M. (2002). Interleukin-6 (IL-6): a possible neuromodulator induced by neuronal activity. Neuroscientist 8, 268–275. 10.1177/1073858402008003012. 12061506

[B28] Kaiser M. Burek M. Britz S. Lankamp F. Ketelhut S. Kemper B. (2018). The influence of capsaicin on the integrity of microvascular endothelial cell monolayers. Int. J. Mol. Sci. 20 (1), 122 10.3390/ijms20010122. PMC6337111 30598013

[B29] Kaneko R. Kato H. Kawamura Y. Esumi S. Hirayama T. Hirabayashi T. (2006). Allelic gene regulation ofPcdh-α andPcdh-γ clusters involving both monoallelic and biallelic expression in single purkinje cells. J. Biol. Chem. 281, 30551–30560. 10.1074/jbc.m605677200. 16893882

[B30] Lajoie J. M. Shusta E. V. (2015). Targeting receptor-mediated transport for delivery of biologics across the blood-brain barrier. Annu. Rev. Pharmacol. Toxicol. 55, 613–631. 10.1146/annurev-pharmtox-010814-124852. 25340933 PMC5051266

[B31] Lampugnani M. G. Zanetti A. Corada M. Takahashi T. Balconi G. Breviario F. (2003). Contact inhibition of VEGF-induced proliferation requires vascular endothelial cadherin, β-catenin, and the phosphatase DEP-1/CD148. J. Cell Biol. 161, 793–804. 10.1083/jcb.200209019. 12771128 PMC2199373

[B32] Lev S. Moreno H. Martinez R. Canoll P. Peles E. Musacchio J. M. (1995). Protein tyrosine kinase PYK2 involved in Ca2+-induced regulation of ion channel and MAP kinase functions. Nature 376, 737–745. 10.1038/376737a0. 7544443

[B33] Lobas M. A. Helsper L. Vernon C. G. Schreiner D. Zhang Y. Holtzman M. J. (2012). Molecular heterogeneity in the choroid plexus epithelium: the 22-member γ-protocadherin family is differentially expressed, apically localized, and implicated in CSF regulation. J. Neurochem. 120, 913–927. 10.1111/j.1471-4159.2011.07587.x. 22092001 PMC3296866

[B34] Maddahi A. Edvinsson L. (2010). Cerebral ischemia induces micro vascular pro-inflammatory cytokine expression via the MEK/ERK pathway. J. Neuroinflammation 7, 14 10.1186/1742-2094-7-14. 20187933 PMC2837637

[B35] Mah K. M. Houston D. W. Weiner J. A. (2016). The gamma-Protocadherin-C3 isoform inhibits canonical Wnt signalling by binding to and stabilizing Axin1 at the membrane. Sci. Rep. 6, 31665 10.1038/srep31665. 27530555 PMC4987702

[B36] Mah K. M. Weiner J. A. (2017). Regulation of Wnt signaling by protocadherins. Semin. Cell Dev. Biol. 69, 158–171. 10.1016/j.semcdb.2017.07.043. 28774578 PMC5586504

[B37] Miralles C. P. Taylor M. J. Bear J. Jr. Fekete C. D. George S. Li Y. (2020). Expression of protocadherin‐γC4 protein in the rat brain. J. Comp. Neurol. 528, 840–864. 10.1002/cne.24783. 31609469 PMC6987019

[B38] Molumby M. J. Anderson R. M. Newbold D. J. Koblesky N. K. Garrett A. M. Schreiner D. (2017). γ-Protocadherins interact with neuroligin-1 and negatively regulate dendritic spine morphogenesis. Cell Rep. 18, 2702–2714. 10.1016/j.celrep.2017.02.060. 28297673 PMC5418859

[B39] Nollet F. Kools P. van Roy F. (2000). Phylogenetic analysis of the cadherin superfamily allows identification of six major subfamilies besides several solitary members 1 1Edited by M. Yaniv. J. Mol. Biol. 299, 551–572. 10.1006/jmbi.2000.3777. 10835267

[B40] Okazaki N. Takahashi N. Kojima S. Masuho Y. Koga H. (2002). Protocadherin LKC, a new candidate for a tumor suppressor of colon and liver cancers, its association with contact inhibition of cell proliferation. Carcinogenesis 23, 1139–1148. 10.1093/carcin/23.7.1139. 12117771

[B41] Peek S. L. Mah K. M. Weiner J. A. (2017). Regulation of neural circuit formation by protocadherins. Cell. Mol. Life Sci. 74, 4133–4157. 10.1007/s00018-017-2572-3. 28631008 PMC5643215

[B42] Phillips G. R. LaMassa N. Nie Y. M. (2017). Clustered protocadherin trafficking. Semin. Cell Dev. Biol. 69, 131–139. 10.1016/j.semcdb.2017.05.001. 28478299

[B43] Sánchez-Elsner T. Botella L. M. Velasco B. Corbí A. Attisano L. Bernabéu C. (2001). Synergistic cooperation between hypoxia and transforming growth factor-β pathways on human vascular endothelial growth factor gene expression. J. Biol. Chem. 276, 38527–38535. 10.1074/jbc.m104536200. 11486006

[B44] Sano K. Tanihara H. Heimark R. L. Obata S. Davidson M. St John T. (1993). Protocadherins: a large family of cadherin-related molecules in central nervous system. EMBO J. 12, 2249–2256. 10.1002/j.1460-2075.1993.tb05878.x. 8508762 PMC413453

[B45] Shalaby M. R. Waage A. Aarden L. Espevik T. (1989). Endotoxin, tumor necrosis factor-α and interleukin 1 induce interleukin 6 production *in vivo* . Clin. Immunol. Immunopathol. 53, 488–498. 10.1016/0090-1229(89)90010-x. 2805453

[B46] Silwedel C. Förster C. (2006). Differential susceptibility of cerebral and cerebellar murine brain microvascular endothelial cells to loss of barrier properties in response to inflammatory stimuli. J. Neuroimmunol. 179, 37–45. 10.1016/j.jneuroim.2006.06.019. 16884785

[B47] Steelman L. S. Chappell W. H. Abrams S. L. Kempf C. R. Long J. Laidler P. (2011). Roles of the Raf/MEK/ERK and PI3K/PTEN/Akt/mTOR pathways in controlling growth and sensitivity to therapy-implications for cancer and aging. Aging (Albany NY) 3, 192–222. 10.18632/aging.100296. 21422497 PMC3091517

[B48] Suo L. Lu H. Ying G. Capecchi M. R. Wu Q. (2012). Protocadherin clusters and cell adhesion kinase regulate dendrite complexity through Rho GTPase. J. Mol. Cell Biol. 4, 362–376. 10.1093/jmcb/mjs034. 22730554

[B49] Wang S. Meng Y. Li C. Qian M. Huang R. (2015). Receptor-mediated drug delivery systems targeting to glioma. Nanomaterials (Basel) 6, 3 10.3390/nano6010003. PMC5302535 28344260

[B50] Wang Y. Chen J. Tang W. Zhang Y. Li X. (2017). Rapamycin inhibits the proliferation of endothelial cells in hemangioma by blocking the mTOR-FABP4 pathway. Biomed. Pharmacother. 85, 272–279. 10.1016/j.biopha.2016.11.021. 27914823

[B51] Weiner J. A. Jontes J. D. (2013). Protocadherins, not prototypical: a complex tale of their interactions, expression, and functions. Front. Mol. Neurosci. 6, 4 10.3389/fnmol.2013.00004. 23515683 PMC3601302

[B52] Wong A. D. Ye M. Levy A. F. Rothstein J. D. Bergles D. E. Searson P. C. (2013). The blood-brain barrier: an engineering perspective. Front. Neuroeng. 6, 7 10.3389/fneng.2013.00007. 24009582 PMC3757302

[B53] Yao J. S. Zhai W. Young W. L. Yang G.-Y. (2006). Interleukin-6 triggers human cerebral endothelial cells proliferation and migration: the role for KDR and MMP-9. Biochem. Biophys. Res. Commun. 342, 1396–1404. 10.1016/j.bbrc.2006.02.100. 16516857

[B54] You C. Erol Sandalcioglu I. Dammann P. Felbor U. Sure U. Zhu Y. (2013). Loss of CCM3 impairs DLL4-Notch signalling: implication in endothelial angiogenesis and in inherited cerebral cavernous malformations. J. Cell Mol. Med. 17, 407–418. 10.1111/jcmm.12022. 23388056 PMC3823022

[B55] Yu J. S. Koujak S. Nagase S. Li C.-M. Su T. Wang X. (2008). PCDH8, the human homolog of PAPC, is a candidate tumor suppressor of breast cancer. Oncogene 27, 4657–4665. 10.1038/onc.2008.101. 18408767 PMC3013056

[B56] Zhang Z. G. Zhang L. Jiang Q. Zhang R. Davies K. Powers C. (2000). VEGF enhances angiogenesis and promotes blood-brain barrier leakage in the ischemic brain. J. Clin. Invest. 106, 829–838. 10.1172/jci9369. 11018070 PMC517814

[B57] Zhou X. Updegraff B. L. Guo Y. Peyton M. Girard L. Larsen J. E. (2017). PROTOCADHERIN 7 acts through SET and PP2A to potentiate MAPK signaling by EGFR and KRAS during lung tumorigenesis. Cancer Res. 77, 187–197. 10.1158/0008-5472.can-16-1267-t. 27821484 PMC5410365

[B58] Zhu H. Dai R. Zhou Y. Fu H. Meng Q. (2018). TLR2 ligand Pam3CSK4 regulates MMP-2/9 expression by MAPK/NF-κB signaling pathways in primary brain microvascular endothelial cells. Neurochem. Res. 43, 1897–1904. 10.1007/s11064-018-2607-7. 30088235 PMC6182369

